# Scientific Intelligence: Recognising It to Nurture It

**DOI:** 10.3390/jintelligence11040060

**Published:** 2023-03-27

**Authors:** Debra McGregor, Sarah Frodsham

**Affiliations:** 1 School of Education, Oxford Brookes University, Oxford OX2 9AT, UK; 2 Department of Continuing Education, The University of Oxford, 1 Wellington Square, Oxford OX1 2JA, UK

**Keywords:** professional scientist, real-world problems, intelligence, originality, science education, learning experience

## Abstract

Successful scientists need to think carefully about the particular aspect of the world around them they are investigating. They build on what is known in their area of science to identify how they might examine the issue or problem they are concerned with to offer further insights. Through investigating natural phenomena, they can solve problems and communicate new ways of looking at the world. Their work serves to address global and societal challenges and often offers improved ways of living. The ways that scientists’ work can have implications for educational processes designed to prepare would-be scientists or scientifically aware citizens of the future. Eliciting reflections from experienced scientists recounting how they came to develop their scientific intellect, expertise and problem-solving know-how is useful to inform science education. This article reports on an aspect of a larger project involving 24 scientists specialising in biological or physical science research from Higher Education Institutions, located in either Manchester, Oxford or London. The study adopts a retrospective phenomenographical methodology and applies two fresh theoretical perspectives to eight in-depth interviews with professional scientists working in university departments involved in ground-breaking research. Conversations with the scientists were framed to explore the nature and extent of formal and informal learning influences affecting the development of their inventiveness and expertise in becoming scientists. The reified perspectives collated here show how a range of experiences have afforded expert scientists the opportunity to apply their intellectual capabilities. These kinds of demonstrable abilities have enabled them to scientifically contribute to being able to solve real-world problems. Additionally, a cross-case analysis of scientists’ reported learning experiences could inform science education policy and practice.

## 1. Introduction

In this paper, we analyse interview data in an exploratory manner. That is, we retrospectively apply two fresh theoretical frameworks to examine the recounted learning and work experiences of eight professional scientists. This has highlighted what they individually and collectively considered relevant to the development of their scientific intelligence, wisdom and know-how. More specifically, the analysis focuses on scientific literacy as articulated through the ways that they have and continue to make investigative decisions based on scientific facts, historical and current research and their own experiences of the natural world that surrounds them; rather than through unsubstantiated opinion and/or hearsay. The analytical frameworks adopted combine various arguments about intelligence, problem-solving and peer collaboration and facilitate consideration and analysis of the ways scientists, working within local and global environments and across micro and macro contexts in physics, experience salient and formative aspects of scientific endeavour. The analysis also explores how these recollected narratives indicate that particular experiences have facilitated and continue to enable successful careers at the cutting edge of scientific research.

### 1.1. Eliciting Scientists’ Recollections of Learning Experience

Currently, we face a range of global crises, including climate change, resource depletion, biodiversity loss, an aging population and the legacy of COVID-19. Prior to the latest pandemic the Organisation for Economic and Co-Operative Development (OECD 2015a, 2015b, 2018, 2021b) initiated various campaigns to raise awareness that promoted ways of learning and revising of educational curricula to develop educational pedagogy that can support and encourage would-be scientists and technologists to collaborate to solve these worldwide issues. Concerned with many policy initiatives, the OECD calls for a Science, technology and innovation culture as well as Research and innovation for society strategy. This strategy targets ‘actors’, such as the scientists interviewed in this study, and within society, from school teachers to members of the general public, whom the OECD suggests could contribute to ‘efforts in addressing societal challenges’ (OECD 2021a). What is omitted from these calls for collaboration is how this could, in reality, be achieved. Indeed, collaboration was central in the race to contain and beat the COVID-19 virus, with the focus being on fostering the expedient development of a safe and effective vaccine(s) (Druedahl et al. 2021). Professional scientists, that is, those who are working at the cutting edge of innovative research akin to vaccine development, are well known for not only being agentive (McGregor and Frodsham 2019) but also working as a global and even local community for the greater scientific good (Sonnenwald 2007). For example, the successful sequencing of the human genome from 1990–2003 was the result of collaboration among numerous scientists from 20 institutions in six different countries (France, Germany, Japan, China, the United Kingdom and America). This was preceded in 1950 by Watson and Crick, with Franklin sharing resources and discovering that the DNA molecule had a double helix structure. Although, admittedly, Rosalind Franklin’s X-ray diffraction images of DNA where not commercially available and were used by Watson and Crick somewhat controversially (Maddox 2002). Franklin’s part in discovering the double helix structure has been posthumously recognised by Maddox. In contrast to the ongoing debate about gender equality in the sciences (Ovseiko et al. 2017), Maddox (2003) stated that her sex was not what made her feel excluded from recognition, but her ‘class and religion’ (ibid:408. Maddox went on to say that Franklin was ‘a wealthy Anglo-Jew [who] felt out of place in a Church of England setting [King’s college] dominated by swirling cassocks and students studying for the priesthood’ (ibid).

Whilst the reciprocal sharing of ground-breaking developments in thinking and problem resolution, no matter what gender you identify as, is arguably not possible during the formative schooling years (ages 4–18), the ways that reciprocal collaboration in science can be promoted at a local level have been evidenced (Fischer and Zigmond 2010; Calabrese Barton and Tan 2010). Whilst this type of scientific endeavour can begin to address societal challenges, long held views about what scientific intelligence is assumed to be should be reviewed to begin to better understand how this can be scaffolded within educational contexts.

### 1.2. Long Held Views of Intelligence 

McGrew et al. (2023) discuss long-established tests that derive ‘general intelligence’ (or measures of ‘g’); however, these overlook important and diverse influences on what constitutes intellect. Sternberg (2022, p. 1) describes how a comprehensive understanding of this phenomenon requires ‘the converging operations of a variety of approaches to it’, rather than understanding it ‘through any one conceptual or methodological approach’ (ibid). These varied operations are considered further below to emphasize the kinds of cognitive functions reportedly adopted by professional scientists when working in specific disciplinary contexts and with their extended communities (Wenger 1998). Sternberg also elaborates that intelligence includes *deciding* how to find something out as well as *thinking critically* about something, including current accepted knowledge. It is these pragmatic ways of thinking (Hedlund 2020) to work out solutions to real-world problems that scientists attend to, and they are salient to the argument(s) in this paper.

### 1.3. Intelligence and Problem Solving

Perkins (2011) recognises intelligence as that which includes experiential and reflective capability. Both of these dimensions of intelligence relate directly to problem-solving. He suggests that experiential intelligence is that which you gain through experience. He describes how it is accumulated as someone navigates their way through various contexts and situations in their life. It may be somewhat serendipitous, as it is not always predictable what we may encounter and be exposed to in the world of work or indeed in an as yet unknown scientific context. Reflective intelligence, Perkins suggests, is also a dimension of intellect that a person might use in the exercising and development of their mental skills (ibid). Somewhat juxtaposed, Sternberg (2020) recognises three facets (his triarchic theory) of intelligence. He suggests that practical intelligence is the ability of someone to successfully function in the environment in which they find themselves; creative intelligence relates to generating innovative suggestions; and analytical intelligence is demonstrated through evaluating data and solving problems. Others, such as Hedlund (2020), also recognise how intelligence is not just cognitive it is also practical. 

Perkins (2011) suggests that through learnable dimensions of intelligence, we ‘can learn to think and act more intelligently’. Goodrich-Andrade and Perkins (2008) describe this as ‘Learnable Intelligence’. Plucker et al. (2020) extend this to demonstrate how creativity or thinking in alternate or original ways is involved in ‘adapting to novel situations that can lead to great success’ (p. 1087) and indeed innovative research and findings. This kind of thinking is beyond the well-established tests that suggest how intelligence is measurable (Kaufman 2015). 

### 1.4. Problem-Solving in Science

Sternberg (1985, 1997) highlights the difference between practical and academic problems. He describes how academic problems tend to be well-defined, formulated by others, and complete in the information they provide. Whereas practical (real-world) problems tend to be poorly defined, may lack the necessary information for a solution and might also be solvable in a wide variety of ways. Solving academic problems and practical problems require different kinds of intellect, as demonstrated by the ways that traditional tests of intelligence (e.g., IQ testing) do not accurately predict success outside academic environments. The human’s adaptability to solve new problems (academic or practical) intelligently, according to Hambrick et al. (2020), as drawn from Stern (1914) and Bingham’s (1937) work, involve planning, reasoning, thinking abstractly and learning from experience. 

**Figure 1:**
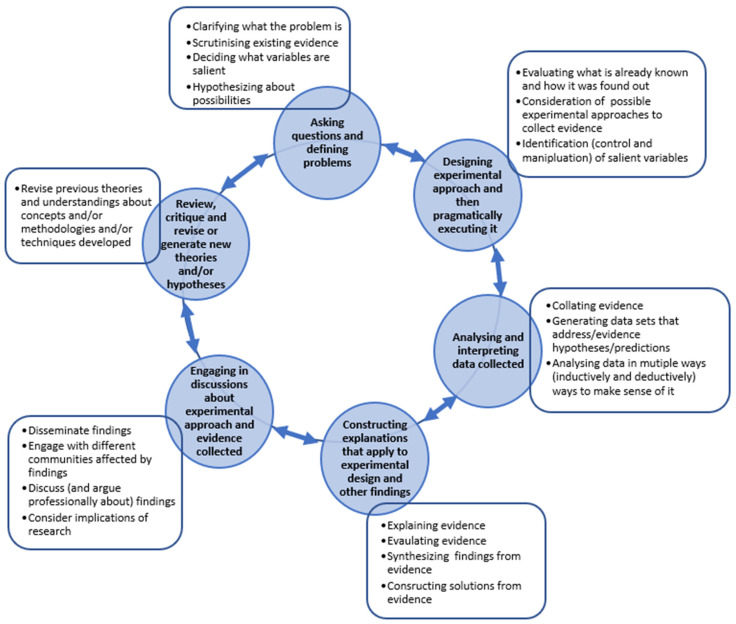
Key features in ill-defined problems (after Mayer 2013; Hambrick et al. 2020) that present opportunities for scientists to apply practical, creative and analytical scientific intelligence (Sternberg 1985) when working toward a solution.

The definition of problem-solving that applies to this study involves the recognition of a desired aim, outcome, or solution where there is no clear pre-determined method to reach a resolution (Hambrick et al. 2020). Scientists are not presented with ‘well-structured problems’ that Mayer (2013) cited in Hambrick et al. (2020, p. 554) refers to. Real-world problems are not resolved by selecting from options offered; for example, the moving discs in the tower of Hanoi challenge can be resolved by avoiding an obvious particular arrangement of the disks. Neither are we thinking about other kinds of low-level tasks where ‘options’ are presented to problem-solvers to choose what they reason is the best solution (such as in multiple choice/Raven’s matrices etc.). Instead, we are concerned with what it is that scientists do to generate solutions to complex real-world (practical) problems. In these situations, options or methods to address a problem or concern are not necessarily obvious. Deciding what matters first (Sternberg et al. 2022) and then ‘figuring out how to’ solve it (Hambrick et al. 2020) is part of the cognitive and pragmatic process to reach a resolution. As Hambrick et al. (2020, p. 573) argue, ‘intelligence can be viewed as the general ability to solve problems’. As such, the junctures (Figure 1) within ill-defined problems (Mayer 2013) are complex (Hambrick et al. 2020) and require non-linear thinking to solve them. This will also require aspects of practical, creative and analytical intelligence (Sternberg 1985).

### 1.5. Problem-Solving in Science and Science Education

Despite the tensions in the way science is taught at school (McGregor and Frodsham 2019) and the processes involved in scientific advances, the OECD (2018) has called for school science to recognise the need for more creative pedagogical approaches to better promote scientific literacy and inquiry aptitudes. This can be reportedly achieved by actively engaging in more questioning in lessons, the development of inquiries, and urging more reflection on ways that problems have been solved through metacognition to support and encourage aspiring would-be scientists (OECD 2015a, 2015b). In 2018, the Educational Endowment Fund (EEF) suggested five reasons to continue to focus on science in schools. The fifth aim, which resonates with the OECD’s (2015b, 2018) campaign, was to ‘open the doors to [a] rewarding and interesting career pupils [who could] aspire to’ (Educational Endowment Fund 2018, p. 1) becoming a scientist at the cutting edge of discovery. However, Durbin, over 40 years ago, suggested that gearing the educational process towards defined objectives, such as performing a specific job, is unwise because the future will be radically different from the world we know today (Durbin 1987). Many organisations now recognise this. The future workplace for current students, however, will change unpredictably, as has been seen by the impact of COVID-19 on the relocation of a vast range of employees working from home. For secondary school students, to achieve occupational success in science, the EEF states, they will have to achieve ‘good’ grades (Educational Endowment Fund 2018). There are many eminent scientists that have, indeed, achieved high academic results during their formative schooling years, e.g., Ada Lovelace (1815–1852), Francis Crick (1916–2004), and more recently, Peter Mitchell (1920–1992). However, there are also many renowned scientists who achieved notable and globally recognised achievements but did not excel during their formative school years. Namely, Michael Faraday (1791–1867), who received little in the way of formal education; Mary Anning (1799–1847), whose family lacked the financial stability to enable her to attend school; Thomas Edison (1847–1931), who only attended school for 12 weeks due to his hyperactive behaviour causing difficulties; and also Jack Horner (1946–present), who found learning extremely challenging at school and fared poorly because of his dyslexia (Mancini 2015). Reflecting on a scientist’s educational experiences, therefore, does not provide the unequivocal evidence that it is only academic performance in school that matters. There are obviously more influences at play.

Eminent scientists, such as those mentioned earlier, have, no matter their educational or socioeconomic backgrounds, thought or worked in innovative ways to conceive of fresh scientific theories, devise new methods or inventions (OECD 2015b, 2018). Creative scientific endeavours have produced revolutionary ideas, including Michael Faraday’s conception of the principles underlying electromagnetic induction, diamagnetism and electrolysis, and Watson, Crick and Franklin’s previously mentioned double helix structure of DNA. It is through these kinds of ingenious scientific discoveries that pressing social and global challenges (such as powering transportation in a carbon neutral way, re-thinking food provision to provide for humanity or recycling plastic materials to minimise energy use and reduce pollution) are met.

### 1.6. Originality in the Science Classroom

The two perspectives of original scientific thinking about everyday life situations or global concepts, such as electrical energy, food production or recycling useful materials not contemplated before, can be considered along a continuum of creativity. At one end, Kaufman and Beghetto (2009) suggest, is mini-c creativity (i.e., when a child initially notices and considers something that has hitherto not been pointed out to them, such as the bubbles on the surface of water before it reaches 100 °C or the extent of water vapour condensed once it has been deemed to have boiled), and at the other end of the big-C creativity spectrum, where an original idea has world-wide significance. These seemingly polar creative positions may seem problematic in the classroom, but unique perspectives emerging between a small group of students or a much larger scientific community sharing their thoughts and interpretations amongst their respective peers can result in personalised expressions of classroom science (McGregor and Frodsham 2019). This can also be extended to worldwide issues through professional scientific forums. McGregor and Precious (2015, pp. 103–25) have demonstrated how aspects of scientists’ work can, to some extent, be mirrored in classroom activities. This can facilitate a deeper level of collaborative scientific problem finding that goes beyond the didactic teaching of scientific facts and strategized prescribed problem solving (Sternberg et al. 2022). As noted earlier in this paper, advances in science were (and are) the combined result of a great many scientists working together (e.g., the human genome project or Watson, Crick and Franklin). Interestingly, Franklin, in a letter to her father, wrote about how her everyday experiences within science propelled her own scientific thinking.

‘…science and everyday life cannot and should not be separated. Science, for me, gives a partial explanation of life. In so far as it goes, it is based on fact, experience and experiment… In my view, all that is necessary for faith is the belief that by doing our best we shall come nearer to success and that success in our aims (the improvement of the lot of mankind, present and future) is worth attaining’

(Maddox 2002, pp. 60–61)

This description of scientific and everyday experience is demonstrable by listening to the student’s explanation as they watch bubbles appearing in heated water. As they watch the water, they begin to formulate their own ideas, bound by their own previous learning experiences. For example, they may have watched a pan of boiling liquid at home, opened a fizzy drink bottle or observed the formation of bubbles as water runs over rocks and stones embedded in a fast-flowing stream. They may formulate a range of abstract explanations from these observations as well as draw from previous experience and knowledge. It is not until they express their interpretations and articulate them for others to understand that we may come to know their unique theorisations.

### 1.7. Science, Scientists and Problem-Solving Intelligence

Franklin’s statement above (Maddox 2002) suggesting the entwinement of the everyday with the scientific resonates with Glück’s (2020) ‘concern for the greater good’ (ibid:1140). She states that the aspiration for future advancement stems from a ‘deep curiosity about the fundamental questions of human existence as well as a willingness to critically reflect’ (ibid). Glück suggests that intelligence is an interwoven component of a larger whole, namely, wisdom. She states that, ‘wisdom integrates the ability to think about complex issues in a complex way’. It is this thoughtful consideration, considering the abstract (theorising from data) as well as the concrete (collating the evidence), with Hambrick et al. (2020) problem solving processes in mind, that informs the scientific process we are concerned with. The process can originate with a query or question about a new phenomenon or something previously unexplained that catalyses scientists to work towards a resolution or comprehension of that inexplicable entity. This resonates with aspects of the Nature of Science (NoS) that Osborne (2014) describes. He argues about the idea of science as a set of practices that include asking questions, developing and using models, constructing explanations, engaging in argument from evidence, planning and carrying out investigation, analysing and interpreting data, using mathematical and computational thinking and obtaining, evaluating and communicating information. 

Alongside these inherent features (see Figure 1) or practices of problem solving within a scientific context, we also adopt Bruner’s (1996) view of the ways that learning involves understanding mental activity within a cultural setting and the resources made available for the learner. As he suggests ‘learning, remembering, talking, imagining, all of them are made possible by participating in a culture’ (Bruner 1996, pp. x–xi). Which also includes learning ‘from experience’, whereby intelligence involves the capability of ‘comprehending surroundings’ and ‘making sense of things’ (Gottfredson 1997, p. 13). Bruner also suggests how ‘the agentive view of mind to be proactive, problem-oriented, attentionally focused, selective, constructional, directed to ends […]. This view encompasses ‘decisions, strategies heuristics […] key notions of the agentive approach to mind’ (Bruner 1996, p. 93). 

This perspective characterises scientific problem-solving in real-world contexts that extend beyond the ‘classic definition of intelligence’ (Darbellay et al. 2022). In summary, this paper suggests that re-thinking the nature of intelligence is needed. Intelligence is not a static, measurable quantity but is concerned with processing complex information (Gottfredson 1997) to resolve conundrums (Hambrick et al. 2020) and explaining phenomena within both local and global contexts. 

## 2. Methodology

This study focuses on the elicitation of scientists’ recounted narratives regarding their individual experiences of scientific endeavour (from their recollected early years through to their current ground-breaking research). Akin to Su et al. (2013), the secondary data used in this study is from a larger qualitative phenomenographic study. 

## 3. Participants

The original data collected involved 60 min interviews with 24 professional scientists from a biological or physical science background. Each had published at least 15 peer-reviewed papers in the last six years and was affiliated with one of three universities (namely, Manchester, Oxford and London). A summary of the eight scientists interviewed and their current focus of work (or role) can be found in Table 1 below (each participant has been de-identified).

## 4. Method: Collecting Data

During the original interviews (papers forthcoming), a second-order perspective was adopted. That is, we invited 24 professional scientists to describe how they were scientifically innovative to gain awareness of their perspective on their scientific endeavours and creativity (Ornek 2008). The in-depth, hour-long interviews framed reflections that phenomenographically suggested how the professional scientists became ground-breaking scholars in science. While we are not the first to interview scientific scholars about their self-evaluations of their creativity using this methodology (Ha and Ha 2022), this paper presents a retrospective phenomenographic analysis of the datasets. Individual narratives of the scientists’ understanding of their intellectual development (Barnard et al. 1999), from an early age through to their current scientific role (at the time of the interviews), were elicited. 

The interview schedule consisted of 13 questions that were framed to explore scientist’s views of their work and their experiences, in and out of school, that impacted their being successful in their previous and current scientific work. The interview approach was semi-structured so that the questions were flexible enough to allow for the scientist to express their own opinion about their experiences and for the exploration of any tangents (O’Leary 2010).

## 5. Method: Analysing the Data

The phenomenographic analysis adopted two distinctive theoretical frameworks (as detailed further below) to examine the narratives of the eight professional scientists in the Oxford cohort. These datasets were scrutinised, both individually and across the cases (Thomas 2017).

With this in mind, the research questions (RQs) posed were:

(1) What do successful professional scientists consider influential in their intellectual development that informs their scientific work?
(2) What does a cross-case analysis suggest about the focus for the development of scientific intelligence?

Each interview was fully transcribed, thematically reviewed in the first instance, and then analysed for the second time by applying two key strands of analysis. The first framework adopts the criteria that Mayer (2013) and Hambrick et al. (2020) recognise as key features of problem-solving. This analysis adopts Saldaña and Omasta’s (2017, p. 120) view of symbolic assignment, which describes how a code in the analysis of qualitative data is ‘most often a word or short phrase that symbolically assigns a summative, salient, essence-capturing, and/or evocative attribute’. Each time the scientists referred to facets of problem-solving, as listed below, a cumulative tally was noted:

- Reasoning (thinking abstractly)
- Solving practical scientific problems (methodological design and experimentation)
- Comprehending (or connecting) complex ideas
- Learning from experience/Experiential intelligence
- Reflectively realising something/reflective intelligence

The second strand of analysis was designed to assess the extent to which the scientists acknowledged that working with others as a member (or participant) of a scientific community notably contributed to their work as scientists. The symbolic assignment (Saldaña and Omasta 2017, p. 120) of references to the different ways that working with others developed their capability as a scientist was collated phenomenographically, akin to Su et al. (2013). The concepts of collaborating as scientists (Cullinane et al. 2022) that we paid attention to were:

- Opportunities to discuss matters scientific;
- Learn through working with others;
- Having opportunities to be agentive (demonstrably proactive and co-constructive with others (Bruner 2008));
- Having opportunities to try out ideas in a supportive way with others.

The application of two theoretical frameworks to yield quantitative data from the analysis of textual information may seem controversial (Maxwell 2010); however, John Platt (1964), a biophysicist and philosopher of scientific inquiry, in his own words stated,

‘you can catch phenomena in a logical box or a mathematical box. The logical box is coarse but strong. The mathematical box is fine-grained but flimsy. The mathematical box is a beautiful way of wrapping up a problem, but it will not hold the phenomena unless they have been caught in a logical box to begin with’ (p. 352).

We have recognised the weaknesses of the logical (the qualitative nature of categorising the data is open to researcher interpretation) and the fragility of the mathematical counts (those who talk more may have more references to specific themes), but the strength of the recollections of scientific intelligence, problem solving and collaboration (Maxwell 2010), as illustrated by experienced professional scientists articulations during their interviews, enables the systematic (quantitative) examination to complement the orientation of the topic being studied (the original phenomenological research study). Simply put, one (the qualitative narrative from the scientists) supports the other (frequency counts of themed instances).

This two-fold quantifiable analysis involving distinct themes was also validated through calculating the interrater reliability (i.e., the reproducibility or consistency of analysis between two reviewers). This was found to be 76% for the first analysis, which related to key capabilities when problem solving and 89% for the second phase of analysis, which examined the different ways the scientists worked with others to develop, and inform their professional careers. Both sets of results are, according to Miles and Huberman (1994), within acceptable levels.

## 6. Limitations

Frameworks, such as those adopted for this paper, may miss crucial aspects of the scientist’s intellectual development, such as how formal or informal early childhood recollections of scientific endeavour did or did not inform future career aspirations. However, alongside forthcoming publications, which will incorporate more about these potentially omitted findings, we have briefly outlined the results of the initial thematic examination of the Oxford cohort below. 

## 7. Findings

### 7.1. A Brief Summary of Original Phenomenographic Research

Commitment to the interviews was exhibited by all interviewees involved in the project, even though they were all busy scientists. It appeared they valued the opportunity to reflect despite being hard pressed for time. Every scientist recounted illustrative examples of all the different forms of intelligence described earlier. These were demonstrable through different problem-solving experiences and were also reified in various forms (as discussed later) within their working contexts. Only four scientists (of the original cohort of 24 from Oxford, Manchester and London) could recall the nature of learning science in primary classrooms but most of them recounted more details about the types of opportunities they experienced and influential teachers they encountered in secondary school. 

### 7.2. Identifying Dimensions of Intelligence That Contribute to Problem-Solving

#### 7.2.1. Abstract Thinking

Each of the scientists within the Oxford cohort recognised that abstract thinking could be reified in various and numerous forms (see Table 2). 

Whilst recounting, rather than quantifying numbers of various accounts of scientific reasoning Sci.1, in defining what and how scientists might think about significant concepts, said,

“…in physics we are grappling with this idea of time. What is time? Actually, we go from one extreme where we say, maybe time doesn’t exist really […]. Fundamentally for atoms at the the quantum level of atomic/subatomic entities, maybe there isn’t any time. But then you have physicists who say, “No, no, actually time is the only fundamental thing”. You know, in ancient Greeks you had Heraclitus, who said that everything changes and so on, “You cannot step into the same river twice”, and you had Parmenides who said, “No, it’s just a static universe. Nothing ever happens really in this universe”. We can still view the universe in both of these ways. They give you the same laws of physics ultimately, but I think when you take that leap of imagination and you say, “Well, maybe time is not there really. Maybe, it’s a derived concept. It’s not fundamental”. You have to make sure that you can somehow make it consistent with with theory of relativity and all the other things that we know.

(Sci.1)

This kind of abstract thinking is summarised by Sci.8, who recognised that what is needed in science is, “an interrogation of of ideas”. Sci.7 extends this to suggest that many scientists are “not satisfied until [they’ve] explained things and got an answer”, just as Sci.2 reiterates, “it is the quest to get the truth but we’re never quite sure whether we’ve got it… it’s an evolving subject. We may get things wrong. We do get things wrong, but that’s part of the process. As long as you just base it on what evidence you have and you’re not making big leaps”. Sci.8 reflects on looking at evidence to suggest how it is scrutinising data that is important to be able to identify, “the things that you didn’t even know you wanted to look at”.

#### 7.2.2. Problem-Solving

Beyond abstract or logical thinking, scientists recognised the role of problem-solving (see Table 3) within their work. Sci.5 described how, “people think it’s all about logic and it is to some extent but it’s also about just having a nose for how to solve a problem”, as Sci.3 further outlined,

“When you’re doing research, looking into something new and you’re trying to understand something new that hasn’t been done before. It’s trying to solve some problem and answer some question. It’s looking at that question or that problem in several different ways [that…] creativity comes in … thinking at the different ways to think about a problem. Different ways to approach something. Maybe you have the sort of straightforward way, but then you might have to be creative and think, “Maybe that doesn’t work” and then, you have to think of other things to do…”.

As Sci.7 suggests, her work is about, “Working out how things work”. This is extended by Sci.8, who reflectively considers the public view of science and describes that,

“Most people, when you see, ‘scientist A says this and scientist B says that’, don’t know how to do anything other than conclude that science is confused. Because they’ve been taught a model where science says ‘A’. […] and it’s really important to give people the skills to get beyond that. Not far beyond it, like, a couple of steps beyond it so that conversation, particularly around health and about the environment, can be more informed”.

This view relates then to the ways that ideas and concepts might be linked or connected. 

#### 7.2.3. Comprehending Complex Ideas

The scientists all indicate how they think about linking and connecting concepts (see Table 4). In describing how complex ideas are related, Sci. 8 describes a particular concern he has recently been thinking about,

“If another galaxy flies past, this galaxy [will] slosh around and… the bar stays fixed and the disc sloshes and then it damps. It’s quite a cool process… The critical bit is, “Well, is it true?” Well one way you might test that is you go looking for the galaxy that’s just flown past. We’ve done that and we can’t find them. So now you’ve got two possibilities”.

Sci.2 explains this a little differently by reflecting on a previous project completed. He says,

I think being in science you’re always pushing against the knowledge boundary… and there are different ways of going about that. Certainly, it’s there’s always something else to do… even though you said, “Hey, look I’ve shown that this agrees with that”, your next job is, so, “Does it agree with something else now?” [Sci.2]

#### 7.2.4. Experiential Intelligence

All the scientists described a range of examples of lived experiences from which they learned something about science (see Table 5). Sci.2 provided insightful details about a practical school experience where he investigated the speed of light,

“A project I did at secondary school, which was in physics, was a project to measure the speed of light in water. As you know, light travels at a very high speed, but it is finite. But then when we talk about the speed of light, what we generally mean is the speed of light in vacuum. But when light travels through a different medium, air to a small degree but water to a greater degree, then it slows down. So, I thought it would be interesting to measure that, which required the construction essentially of a long water pipe with glass ends, and then setting up a very fast motor that span at very high speed with a little mirror on it, so you could send the light backwards and forwards and measure it. It sort of worked, eventually”.

#### 7.2.5. Reflective Intelligence

The scientists did not so readily describe learning through reflection (see Table 6), as it appeared to be critical reflection over a longer period of time that reified wisdom as an ability to think about complex issues (Glück et al. 2022), whereas learning directly from experience (Table 5) appeared to be more immediately recognisable. As Varshey and Barbey (2021) argue metacognition, thinking about the cognitive processes or operations that have been applied, can affect mental procedures (Bruner 2008, p. 128) adopted later. Many of the scientists observed that what was taught to them in school was about learning (limited versions of Bohr’s or Boyle’s Law (Sci.1) for example) and being able to remember and recite it for examinations. A common theme, however, was that which Sci. 4 described spending more time as scientists contemplating what something meant than learning [prescribed] science as a student in school.

“One of the most important things that people do is to collect their data and then spend some time looking at it, and perhaps presenting it in different ways. So, if you’ve got an image, you can present it as a two-dimensional map, and you can break it down and look at different regions and see how they compare. You can look at the properties of individual objects in the image, and you can look for connections between them. So, there’s lots of ways of looking at data that may give you different kind of information. I think having the time to play, [that] isn’t quite the right word, but to use different[ly], to take different viewpoints”.

(Sci.4)

Interestingly, it appeared that experiential learning—the opportunity to learn through manipulating objects, equipment and data—was foremost in the scientist’s minds. This relates to Perkins (2011) view that as people, in this case scientists, navigate their way through the various opportunities in their lives, they utilise the opportunities to develop their experiential intelligence. The data in Table 7, also indicates the frequency and range of references by the eight scientists to the different components of experiential intelligence that could be honed through involvement in problem-solving. 

#### 7.2.6. Developing Thinking through Discussion, Interacting and Working with Other Scientists

It was notable that beyond just eliciting individual skills or capabilities that scientists themselves recognised they employed in their work, they also realised how important working with other scientists was. 

All the scientists, within their 70-min conversations, recounted more than 200 incidents where others were important in their work (see Table 8). The recollections included discussions with other scientists that involved engaging in arguments with those holding opposing views or degrees of scepticism regarding a current experimental approach or set of findings. This is exemplified by Sci. 8, who says, “the best days are the ones when I walk down the hall, have an argument with somebody about whether black holes affect the galaxies”.

Other scientists also valued collaborations (see Table 8) with groups of like-minded scientists as well as their Masters and PhD students, who might question assumptions, present confusing data, or interpret evidence in alternate ways. Sci.8 said,

We’ve also got a master’s student, who’s discovered that lots more galaxies are lop-sided than we thought. This week [we’re] trying to work out what that means”.

Sci.7 indicated how she felt that,

“…within that group of scientists that I was working with [as a postgraduate], it was really fun, really creative. … I was incredibly lucky because I ended up doing a PhD for […] a Government Chief Scientist who really publicised climate change”.

Sci.6 indicated that he,

“became an experimentalist because I really like working in international teams. Not just working in the ivory tower by myself, but working with other people, and I worked in the early ‘90s at CERN. I was a summer student there. What intrigued me most was we worked with people at the time, with Americans, Russians, Chinese, all working on the same experiment together”.

The cultural context of scientists working collectively on a shared or common problem, appears to matter where practical problem-solving intelligence is required and tacit knowledge is salient. In these situations, tacit knowledge is shared by working together and pooling insights from experience and know-how. Sternberg (2020) suggests that this kind of cultural influence on intelligence is as yet under-researched and a potential area for future investigation. 

Leading and being agentive were described by Sci. 2, who said,

“I think part of the skill for someone in my position now is to see what the strengths and the weaknesses of the people who I employ or my PhD students are and help them to work in the best way. If you get someone who’s working their optimal way, you both benefit way more because more gets done and you have usually have way more ideas passed between you because you’re giving them the freedom to open up a little bit”.

(Sci.2)

As Sci.3 indicated, though, having opportunities where others can scrutinise and critique your work is crucial. He said,

“I think that’s crucial for our field probably it’s really important in any field. It’s getting that feedback. There are so many little details in this research that you can miss. You really have to discuss it with someone and make sure you haven’t missed any detail. You may miss something. You may not have that particular creative light that someone else has”

(Sci.3)

## 8. Discussion

This retrospective phenomenographic analysis of scientists’ recollections of the kinds of intelligence they needed to succeed in their work (and thereby answer RQ1) has shown how a range of experiences have afforded them opportunities to develop diverse but interwoven capabilities. It is these that have contributed to being able to solve real-world problems. The various capabilities include reasoning or thinking abstractly, solving practical scientific problems (devising/designing methods/approach to answer query), comprehending (or connecting) complex ideas, experiential intelligence and reflective intelligence (as presented in Table 2, Table 3, Table 4, Table 5 and Table 6). The scientists recognised that to engage in successfully solving practical real-world problems, they needed unconstrained opportunities to investigate; opportunities to *think*, *explore* and *do things* independently; working with others mattered; flexibility and agility of (abstract) thought; tenacity and opportunities to demonstrate agency (being proactive and co-constructive). They were all, at some point, working agentively in a way that was problem-oriented, focused, selective, constructional and directed to ends (Bruner 1996). These kinds of cognitive and practical enactments were evident from the recollections of scientists’ experiences, but are not necessarily recognised or supported in school science (McGregor and Frodsham 2019). Whether the goal of science education is to develop capabilities to become a scientist or to be an informed citizen, aspects of science education could be better developed to offer more appropriate opportunities for students to practice or rehearse the kinds of intellectual abilities required to work as a scientist. In answering RQ2, the cross-case analysis suggested that educational experiences should relate to the various components of intelligence that scientists need to apply in their work. This will include the way that science is presented to students. Tala and Vesterinen (2015) suggested that science needed to be considered in different contexts and from different viewpoints. This was also noted by the scientists in this study. One scientist suggested that being made aware of how observations and theory relate was important. He described how,

“there was one inspirational teacher who… started with Newton’s Laws… and she showed us how to derive a certain relationship in thermodynamics…So actually, you can derive this ideal gas law but starting from very primary concepts such as atoms moving about, colliding and and then applying statistics to this and out comes a fundamental law of thermodynamics. And I thought this was really mind blowing… You’re able to actually predict what happens at the microscopic and and I thought, you know, these connections are typical for physics. That you’re connecting things… I think this was the first instance maybe when I saw that that you can use the micro description to derive the macro description”

(Sci.1)

The contexts suggested by several scientists were alternate ways teachers could extend opportunities for learners to explore through experience and discussion with experienced others.

“I went there [my old primary school] with a talk I thought would be good and a few things to get them to do, and I just started talking to them and they just kept asking me questions for an hour. It was just like, “Oh”, and I didn’t get onto any of the things I prepared. I just talked to them, and they were just really excited and really keen to have someone who could hopefully speak their language who comes from somewhere else that isn’t their teacher”

(Sci.2)

Experiencing science is not just about the doing; it is also about the explanatory, abstract thinking, as two other scientists indicated,

“He [the secondary school physics teacher] would have us do quite a lot of experiments where he would say, “Let’s do this experiment”, and then, you know, “What is happening?”

(Sci.3)

Recognising how school science does not extend opportunities that directly relate to doing real science is also important,

“I guess the one thing I didn’t realise when I was in secondary school was the creative aspect of science because there’s actually very little opportunity to understand, well, how has a scientist come up with this idea? Or how has an experimentalist or an observer come up with the the way of testing this idea? I never found there was that kind of scope when I was at school, and now as a research that’s basically all I do”.

(Sci.2)

Teachers offering opportunities to demonstrate agency is also pertinent, as Sci.86 illustrated,

“At Sixth Form, there were a small group of us who were sort of very interested in this this stuff and we were given a lot of space to to ourselves, outside, you know, literally given—or a tolerated coming out in the lab like at lunchtime and so on—and that gave us the space to talk and to argue about these things”

(Sci.86)

Sci.86 also recognised how the content of school science is different,

“I think school science, for me, in the classroom, the joy was supposed to be getting the right answer. I think a lot about experiments with ticker tape and stuff where you’re measuring ‘g’, right, and you sort of have a sense of how well you did because we know that ‘g’ is supposed to be 9.81 and so if you get 9 that’s pretty good, if you get 9.8 that’s even better. That’s just not where the joy is for me”.

(Sci.86)

The nature of school science learning and the nature of scientists’ work remain distinctly different (Tala and Vesterinen 2015), but it is possible that teachers (and schools) can generate appropriate opportunities for students to practice thinking abstractly, engaging in discussion about scientific matters, and enacting agentive practical problem-solving. Opportunities could be offered beyond the science classroom so that students could learn through working with professional scientists, such as those interviewed in this study. 

## 9. Conclusions

There continue to be tensions between policy influences and opportunities for students to develop agency in school science that need addressing (McGregor and Frodsham 2019). As indicated by the scientists’ comments, agentive and open opportunities to experiment, problem solve and work collaboratively in school are limited. However, scientists clearly recount the kinds of intellectual capabilities required to be scientists in the real world. This paper suggests beyond the OECD (2018, 2021a, 2021b) what educators should pay attention to to provide more realistic and authentic science learning experiences in school. 

The findings are fascinatingly insightful and offer suggestions about the ways that educational experiences centred around practice in resolving ill-defined problems or inquiries could better support the development of scientific intelligence (i.e., by thinking beyond what can be measured—IQ and the attainment grades obtained during schooling years). Ovenden (2019, p. 27), a marine scientist from Australia, stated ‘there is no definite recipe for success’. Indeed, scientists come from all walks of life and have had a variety of social, cultural and historical experiences (as is the case for the eight interviewed for this paper). However, Ovenden went on to say ‘being great at science is not enough. To be “great”, a lot of other skills [are] needed’ (ibid:24). We have highlighted how this involves interpersonal collaborations, not just between national and international scientific colleagues but also in more informal settings with family members and friends. These facilitated the scientists thinking abstractly, systematically, and reflectively. Ovenden called this being ‘mentally flexible’ (ibid) and it is this that has been essential for all eight scientists’ successful careers. Consideration of the views of these experienced and successful scientists can inform science education policy and practice. Educational practices could be re-oriented from being attainment driven to becoming, and continuing to think like a scientist; this could better prepare aspiring scientists or our future informed citizens.

This cross-case analysis suggests how a scaled-up study could elaborate further on key learning experiences that are essential in educational policies and practices that could better enable would-be future scientists to develop their curiosity, inventiveness and a flair for thinking flexibly through both creativity and critical thinking. 

## Figures and Tables

**Table 1:** Participants’ geographic and demographic data.

Location	Scientist De-Identified Designation	Country/Place of Education	Current Role	Number of Published Articles in the Previous Six Years
Oxford	Sci.1	Serbia	Quantum physicist	70
	Sci.2	England	Astrophysicist	158
	Sci.3	America	Quantum physicist	649
	Sci.4	England	Astrophysicist	21
	Sci.5	England	Oceanic and Planetary Physicist	35
	Sci.6	Germany	Neutrino physicist	61
	Sci.7	England	Material scientist	18
	Sci.8	England	Astrophysicist	71

**Table 2:** Scientists’ references to their abstract thinking.

Scientific Thinking Is Identified as …	Frequency Count per Scientist							
	Sci.1	Sci.2	Sci.3	Sci.4	Sci.5	Sci.6	Sci.7	Sci.8
Reasoning ([=thinking abstractly)	7	4	11	7	6	9	7	20

**Table 3:** Scientists’ references to problem-solving.

Scientific Thinking Identified as…	Frequency Count per Scientist							
	Sci.1	Sci.2	Sci.3	Sci.4	Sci.5	Sci.6	Sci.7	Sci.8
Solving problems (devising/designing methods/strategy)	7	5	15	8	7	9	10	22

**Table 4:** Scientists’ references to comprehending abstract ideas.

Scientific Thinking Identified as…	Frequency Count per Scientist							
	Sci.1	Sci.2	Sci.3	Sci.4	Sci.5	Sci.6	Sci.7	Sci.8
Comprehending (or connecting) complex ideas	10	11	13	10	5	7	6	19

**Table 5:** Scientists’ references to experiential intelligence.

Scientific Thinking Identified as…	Frequency Count per Scientist (Sci)							
	Sci.1	Sci.2	Sci.3	Sci.4	Sci.5	Sci.6	Sci.7	Sci.8
Learning from experience/Experiential intelligence	10	11	23	9	18	11	15	3

**Table 6:** Scientists’ references to reflective intelligence.

Scientific Thinking Identified as…	Frequency Count per Scientist (Sci)							
	Sci.1	Sci.2	Sci.3	Sci.4	Sci.5	Sci.6	Sci.7	Sci.8
Reflectively realising something/reflective intelligence	7	7	5	3	3	4	2	12

**Table 7:** Contrasting the different facets of intelligence as reflected upon by the eight Oxford scientists.

Scientific Thinking and Acting Identified as…	Average	Range
Reasoning	8.9	4–20
Solving problems	10.4	5–22
Comprehending	10.1	5–19
Experiential intelligence	12.5	3–23
Reflective Intelligence	5.4	2–12

**Table 8:** Scientists’ references to developing intelligence through working with others.

Working as a Participant in a Scientific Community	Frequency Count per Scientist (Sci)								Cum. Total
	Sci.1	Sci.2	Sci.3	Sci.4	Sci.5	Sci.6	Sci.7	Sci.8	
Opportunity to discuss scientific matters	11	8	14	3	7	8	7	18	76
Learning through working/participating/developing experience with more experienced scientists	13	13	20	8	14	10	12	11	101
Having opportunities to be agentive (being proactive/co-constructive/reflective with others)	10	8	10	5	7	5	3	9	57
Having opportunities to try out ideas/have others critique in supportive way your work	4	5	11	3	4	7	3	4	41

## Data Availability

Data is not publicly available.
